# Estimating the velocity of chemically-driven Janus colloids considering the anisotropic concentration field

**DOI:** 10.3389/fchem.2022.973961

**Published:** 2022-08-12

**Authors:** Lijie Lei, Rong Cheng, Yuxiu Zhou, Tiezhu Yang, Beirong Liang, Shuo Wang, Xinyuan Zhang, Guanhua Lin, Xuemao Zhou

**Affiliations:** ^1^ College of Aviation Engineering, Civil Aviation Flight University of China, Guanghan, China; ^2^ School of Mechanical and Electrical Engineering, Guangxi Science and Technology Normal University, Laibin, China; ^3^ Julong College, Shenzhen Technology University, Shenzhen, China; ^4^ Institute for Advanced Study, Shenzhen University, Shenzhen, China

**Keywords:** active colloids, Janus colloids, finite element analysis, Legendre expansion, size effect

## Abstract

The application of the active colloids is strongly related to their self-propulsion velocity, which is controlled by the generated anisotropic concentration field. We investigated the effect of this anisotropy on velocity induced by numerical treatments and size of Janus colloids. The far-field approximation is effective in estimating the velocity, even though it neglects the shape effect on the anisotropy of the concentration field. If the surface mobility contrast between the active and the inert part is moderate, the spherical approximation is feasible for sphere-like Janus colloids. Legendre expansion of the concentration field causes artificial anisotropy. Raising the order of the expansion can suppress this effect, but also distorts the concentration field at the top of active part. Thus, the order of the expansion should be chosen carefully depending on the goal of the study. Based on the verified Legendre expansion method and ionic-diffusiophoresis model, we show that due to the size-effect on both the concentration field and the surface mobility, increasing size of colloids can lower the self-propulsion velocity. Our finding is consistent with previous experimental observations without fitting parameter, shedding new light on the self-propulsion mechanism of chemically-driven active colloids. We further show a velocity reversal at high overall *ζ* potential induced by increasing size, providing a new way for controlling the dynamics of acitve colloids.

## Introduction

Synthetic microswimmers that transfer energy from the environment into their self-propulsion, also known as “active colloids” or “micromotors”, have shown promising potential in biomedical application, environmental remediation and micro-fabrication (Li et al. (2014); De Ávila et al. (2017); Wang et al. (2019); Hortelao et al. (2021); Tong et al. (2021); Urso et al. (2021)). They also play an important role as a model system in studies of soft matter and non-equilibrium physics (Solon et al. (2015); Gompper et al. (2020); Liebchen and Mukhopadhyay (2022)). One of the often encountered micromotors is the chemically driven Janus colloid, which stimulates chemical reactions at the active face. With the activity contrast between the active and inert face, the resulting chemical concentration field is anisotropic, so that the colloid self-propels due to the concentration gradient (Golestanian et al. (2005); Gibbs (2020)). Without the anisotropy of the concentration field, the velocity of the Janus colloids vanishes, leaving only the inter-particle interaction to manifest the colloids’ activity (Soto and Golestanian (2014)). Therefore, understanding how the anisotropic concentration field affects the velocity of micromotors quantitatively is critical from both a fundamental and an application perspective.

The anisotropy of the concentration field is controlled by the angular distribution of the surface activity (or surface flux). Therefore, a colloid’s shape is important in determining its self-propulsion velocity. Although non-spherical shapes can be found in a variety of experimental systems (Brooks et al. (2019); Baker et al. (2019); Gibbs (2020); Sun et al. (2020); Shah et al. (2020); Zhu et al. (2021); Sun et al. (2022)), the spherical approximation based on the Legendre expansion method is still widely applied in theoretical studies (Golestanian et al. (2007); Bianchi et al. (2013); Papavassiliou and Alexander (2017); Campbell et al. (2019); De Corato et al. (2020)) due to the lower computational cost compared to resolving the shape using techniques such as the finite element method (FEM) (Lyu et al. (2021); Zhou et al. (2021)) or the boundary-element method (Varma et al. (2018); Baker et al. (2019)). Far-field approximation is also employed to further improve calculation efficiency, especially when adapting to simulate a large number of colloids (Soto and Golestanian (2014); Lei et al. (2022)). However, the quantitative validation, even for the nearly-spherical colloids, is still lacking. Moreover, the Legendre expansion can lead to artificial anisotropy in the concentration field, requiring higher-order (up to the order of 5) expansion to fit the experimental observations (Campbell et al. (2019)). This artificial anisotropy will also affect the prediction of the pair-wise interaction (Saha et al. (2019)). Therefore, validation of the Legendre expansion method is essential to lay the foundation of its application. The recent development of the theoretical model of ionic-diffusiophoresis quantitatively connects the *ζ* potential of colloids and the ionic strength of the solvent to the velocity of Janus colloids (Zhou et al. (2021)). It is therefore possible to experimentally examine the effect of anisotropic concentration field on the self-propulsion velocity and verify the Legendre expansion method.

On the other hand, the velocity of a Janus colloid can be designed by patterning the surface activity (*α*) and the mobility (*μ*) (Golestanian et al. (2007)). Hence, the anisotropy of the concentration field is crucial for this designing framework. This framework also suggested that the velocity of Janus colloids is independent of their size (Golestanian et al. (2007)), yet the later experimental study showed the opposite, i.e., the velocity decreases drastically as the size increases (Ebbens et al. (2012)). To date, the mechanism of the size-dependent velocity has been under debate for a long time (Ebbens et al. (2012); Brown and Poon (2014); Brown et al. (2017)). Size effect on the velocity was indicated in the result ionic-diffusiophoresis model. In our previous work, we focused on the externally driven motion of passive colloids which was investigated using the far-field approximation (Zhou et al., 2021). Upon verifying the Legendre expansion method, further study on the size effect on the self-propulsion velocity considering the anisotropic concentration field is needed.

In this paper, based on the velocity results of TPM/haematite Janus colloids and the ionic-diffusiophoresis model, the spherical approximation of nearly-spherical Janus colloids and the artificial anisotropy produced by the Legendre expansion method were validated, so that the size effect on the self-propulsion velocity can be investigated. We aimed to provide an alternative mechanism for the size-dependent velocity previously observed on Pt/polystyrene system, and shed new light on the self-propulsion mechanism of chemically-driven Janus colloids.

## Methodology

### Ionic-diffusiophoresis

The activity of the surface of a colloid generates an anisotropic concentration field, whose gradient creates forces on the solvent in the vicinity of a colloidal surface only, leading to a diffusiophoretic solvent flow across the colloid’s electric double layer, determined by the surface mobility distribution. This flow, in turn, causes a net motion of the colloid opposite to the surface-averaged solvent flow, preserving the colloid’s and solvent’s total momentum (Prieve et al. (1984); Liebchen and Mukhopadhyay (2022)). In the framework of the ionic-diffusiophoresis theory, the self-propulsion velocity of a colloid is given by (Zhou et al. (2021)):

$$\vec{v}=\mu \nabla \ln c_{\text{g}}\;,$$ (1)

where *μ* is the surface mobility and *c*_g_ is the concentration of the generated ions. We consider the small *ζ* regime $\left(|\bar{\zeta }|<4\right)$ ,

$$\mu =\frac{\epsilon }{4\pi \eta }{\left(\frac{k_{\text{B}}T}{Ze}\right)}^{2}\left[\beta \bar{\zeta }\left(1-3\lambda \right)+\frac{1}{8}{\bar{\zeta }}^{2}\left(1-\frac{21}{2}\lambda \right)\right]\;,$$ (2)

where $\bar{\zeta }=Ze\zeta /k_{\text{B}}T$ is the normalised *ζ* potential, *ϵ* is the permittivity, *η* is the viscosity of the solution, *k*_B_ is Boltzmann’s constant, *T* is the temperature, *Ze* is the charge carried by a single ion, $\beta =\frac{D_{+}-D_{-}}{D_{+}+D_{-}}$ is the difference between the diffusion coefficient of generated cations and anions. The ionic strength of the solution is linked to the mobility through the Debye Length $\kappa^{-1}={\left(\epsilon k_{\text{B}}T/\sum_{l}z_{l}^{2}e^{2}c_{l}\right)}^{1/2}$ normalised by the radius of the colloid *a*, i.e. *λ* ≡ 1/*κa*, where *l* = *b*, *g* representing the background and generated ions, respectively.

### Finite element method

The finite element method (FEM) is used to resolve the anisotropy of the concentration field of produced ions while taking the shape of the Janus colloid into account. In an infinite fluid domain, the concentration field is given by the steady state diffusion equation:

$$\nabla \cdot \left(D\nabla c\right)=0\;,$$ (3)

where $D=\frac{2D_{+}D_{-}}{D_{+}+D_{-}}$ is the diffusion coefficient of the electrolyte. The concentration gradient at the particle surface is determined by the surface flux *J*:

$$-Dn\cdot \nabla c\left(r_{\text{s}}\right)=J\left(r_{\text{s}}\right)\;,$$ (4)

where **r**_**s**_ denotes the colloidal surface and *J* is set as *J*_0_ ≠ 0 and 0 at the catalytic and inert part of the surface, respectively. The far field boundary condition of the concentration is set to 10^–4^ mM (from the self-ionization of water). The concentration gradient generates a slip velocity at the interface of the particle/solution determined by Eqs 1, 2. The incompressible fluid (∇ ⋅**u** = 0) is driven by the stokes equation, − ∇*P* + *η*Δ**u** = 0, where *P* is the pressure. The concentration and flow fields are then solved numerically. The self-propulsion velocity of the colloid is given by surface-average of the fluid flow along the polar component ${\hat{e}}_{\text{z}}$ (pointing to the inert face) with the opposite direction, i.e. $v=-\frac{1}{4\pi a^{2}}\int ds\;v\left(r_{\text{s}}\right){\hat{e}}_{\text{z}}$ . The Stokes equation was then solved numerically by requiring the particle is force free and obtain the self-propulsion velocity *v*.

### Legendre expansion method

Here, we consider a spherical Janus colloid of radius *a* which is azimuthally symmetric with surface properties depending only on the ‘latitude’ angle *θ* with the polar axis ${\hat{e}}_{\text{z}}$ . We can expand the concentration field in Legendre polynomials *P*_*n*_(cos *θ*), *n* = 0, 1, 2, ⋯ (Golestanian et al. (2007); De Corato et al. (2020)). The surface flux $J\left(\theta \right)=\sum_{0}^{\infty }J_{n}P_{n}\left(\cos\theta \right)$ induces a concentration field according to Eqs 3, 4, so that (Ibrahim and Liverpool (2015)):

$$c\left(r,\theta \right)=c_{\infty }+\frac{a}{D}\overset{\infty }{\underset{n=0}{\sum }}\frac{J_{n}}{n+1}{\left(\frac{a}{r}\right)}^{n+1}P_{n}\left(\cos\theta \right)\;.$$ (5)

**FIGURE 1:**
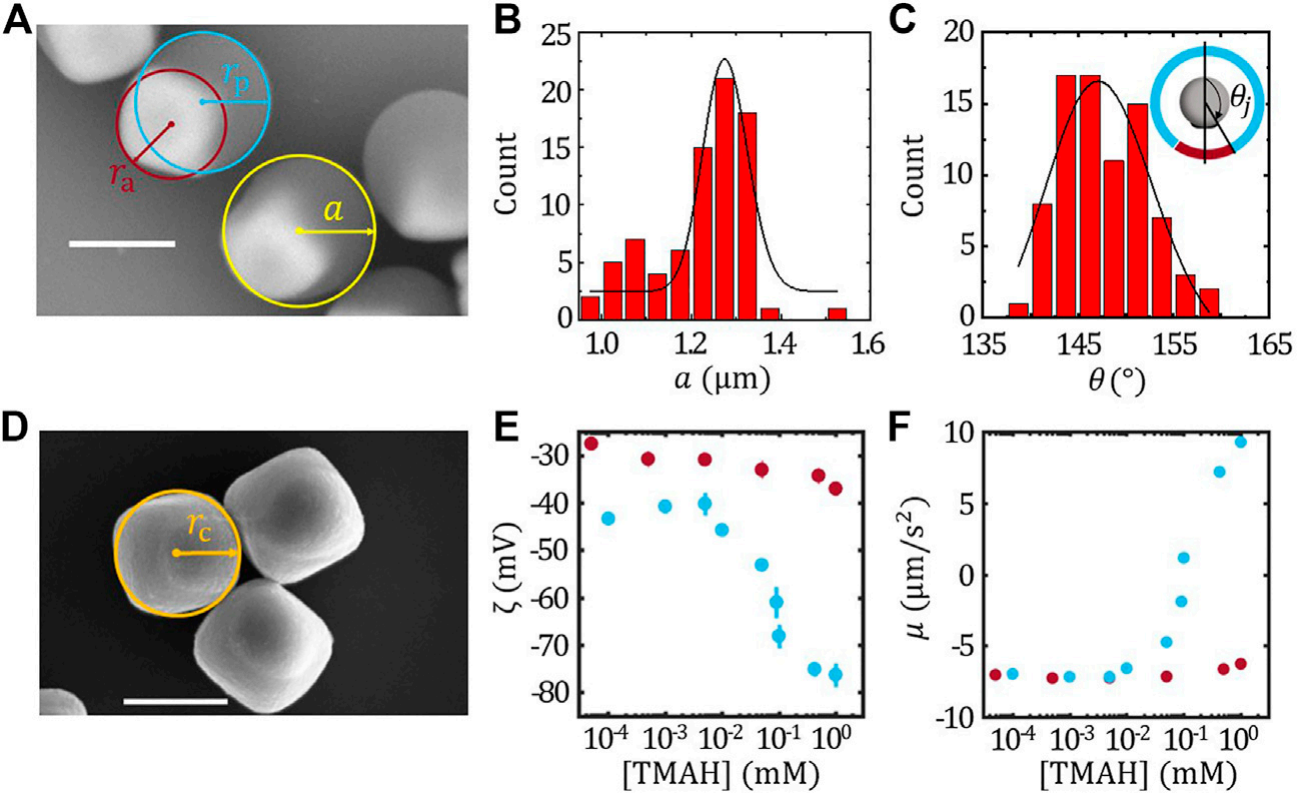
Characterization of TPM/haematite Janus colloids. **(A)** The SEM image of the TPM/haematite Janus colloids and the definition of radius of catalytic (*r*_a_), inert (*r*_p_) part and the radius of the Janus colloids **(A)**. Statistics of the radius of Janus colloids *a* **(B)** and the joining angle *θ*_j_ **(C)**. The inset of **(C)** illustrates the definition of *θ*_j_. **(D)** SEM image of the haematite colloids and the definition of the equivalent radius of haematite *r*_c_. The *ζ* potential **(E)** and the surface mobility **(F)** of the haematite (red) and TPM (blue) colloids. Scale bar = 1 μm.

Therefore, according to Eq. 1, the velocity of the Janus colloid is given by (Tătulea-Codrean and Lauga (2018); Saha et al. (2019)):

$$v=-\frac{1}{4\pi a^{2}}\int ds\;v\left(r_{\text{s}}\right){\hat{e}}_{\text{z}}=-\frac{1}{2a}\int_{0}^{\pi }\frac{\mu \left(\theta \right)\sin\left(\theta \right)}{c\left(a,\theta \right)}\frac{\partial c}{\partial \theta }{\hat{e}}_{\text{z}}d\theta \;,$$ (6)

where *μ*(*θ*<*θ*_j_) = *μ*_a_ and *μ*(*θ* ≥*θ*_j_) = *μ*_p_ are the surface mobility of catalytic and inert face, respectively, and *c* (*a*, *θ*) is the ionic concentration at the colloidal surface. In the calculation, the angular resolution is *dθ* = 1° if not specified. The joining angle *θ*_j_ characterizes the size of the catalytic surface as illustrated in Figure 1, which is determined by the experimentally measured radius of the catalytic (*r*_a_) face and the Janus colloid *a* as sin (*θ*_j_) = *r*_a_/*a*.

### Experimental section

To verify the theoretical treatments, we considered a model system of Janus colloids consisting of a haematite cube partially encapsulated by a TPM sphere. Haematite cubes were synthesized by the sol-gel method introduced by Sugimoto et al. (Sugimoto et al. (1993)). Briefly, in a 250 ml Pyrex bottle, 90 ml of 6 M NaOH (puriss, Sigma-Aldrich) was mixed thoroughly with 100 ml of 2 M FeCl_3_.6H_2_O (p.a., Sigma-Aldrich). Then 10 ml of DI water was added and the resulting gel was left undisturbed at 100 °C for 8 days. Next, the particles were washed several times with DI water. The final product was dried and re-suspended to obtain 0.0125 g/ml aqueous suspension.

TPM/haematite Janus colloids were synthesized according to a modified protocol (Youssef et al. (2016)). First, 1 ml of 3-trimethoxysilyl propyl methacrylate (TPM, ≥98% Sigma-Aldrich) was hydrolyzed (named as hTPM) by magnetically stirring it with 10 ml DI water in a 30 ml glass vial. Then 1 ml of hTPM was added to 13.0 ml DI water containing 0.1 ml cube-suspension and 3.4 μL of ammonia solution (25% wt., Sigma-Aldrich). The mixture was left undisturbed for 30 min. Next, the TPM was polymerized by addition of 10 mg 2,2′-Azobis (2-methylpropionitrile) (AIBN, 98% Sigma-Aldrich) and leaving the glass vial in a pre-heated oven at 80°C for 3 h. The particles were collected by centrifugation at 1,200 rpm for 10 min and washed with DI water for 10 times.

The self-propulsion velocity of TPM/haematite colloids were systematically measured in H_2_O_2_ aqueous solutions with varying concentration of tetramethylammonium hydroxide (TMAH). The details of the experiment can be found in our pervious paper (Zhou et al., 2021). To further study the effect of anisotropic concentration field on the self-propulsion velocity, the surface mobility, and thus the *ζ* potential, of both catalytic and inert part of the Janus colloids should be determined individually. The purified TPM and haematite colloids were dispersed in DI water or TMAH solutions with concentrations matching the [TMAH] in the velocity measurements. The *ζ* potential measurements were performed using a Zetasizer (Brookhaven Instruments NanoBrook 90PLus PALS).

## Results and discussion

Firstly, to determine the parameters for constructing simulation models, we characterise the TPM/haematite colloids. Secondly, the shape effect on the velocity will be examined by comparing the far-field approximation and FEM results with the experimental observations. Thirdly, the Legendre expansion method will be interrogated using the benchmark of concentration field obtained from the experimentally-verified FEM model. Finally, we extend the ionic-diffusiophoresis model to the widely studied Pt/Polystyrene (PS) model system and investigate the size effect on the self-propulsion velocity using the verified Legendre expansion method and FEM.

### Characterization of TPM/haematite Janus colloids

The geometry of FEM model and the Legendre expansion method was constructed based on the size of each component of the TPM/haematite colloids. Figure 1A shows the SEM image of TPM/haematite colloids. The radius of TPM/haematite Janus colloids *a* can be determined statistically as shown in Figure 1B, giving *a* = 1.16 ± 0.01 μm. Combined with the EDX result (See SI), the haematite part and TPM part of the Janus can be identified, and thus the radius of catalytic part *r*_a_ and the inert part *r*_p_ can be measured, giving *r*_a_ = 0.62 ± 0.01 μm and *r*_p_ = 0.88 ± 0.01 μm, respectively. The joining angle *θ*_j_ were also measured and the histogram of *θ*_j_ is shown in Figure 1C together with the Gaussian fit, giving *θ*_j_ = 147.3 ± 0.8°.

We have also measured the equivalent radius of the haematite cube *r*_c_ as illustrated by the cycle in the SEM image Figure 1D, giving *r*_c_ = 0.64 ± 0.01 μm, which was slightly larger than *r*_a_. Considering that the haematite particles are cubic shaped, this result suggests that the exposed part of the haematite in the Janus is mainly the facet of the cube rather than the vertex. Therefore, the detail of the cubic shape can be neglected in the FEM model.

Furthermore, according to Eq. 2, the *ζ* potential of the haematite *ζ*_a_ and TPM *ζ*_p_ colloids determines the surface mobility of the active and inert face, respectively. Therefore, *ζ*_a_ and *ζ*_p_ as a function of [TMAH] matching with the velocity measurements were characterised, and thus the corresponding surface mobility of the haematite (*μ*_h_) and TPM (*μ*_TPM_) colloids were calculated assuming the surface flux *J*_0_ = 2.2 × 10^–3^ mol/(m^2^ ⋅s) as determined from fixed haematite experiment (Zhou et al., 2021). The results were plotted in Figures 1E,F, respectively. It is found that *ζ*_a_ is insensitive to the [TMAH], while *ζ*_p_ varies drastically as [TMAH] >0.01mM .

### The effect of the colloidal shape

The far-field approximation considers an isotropic concentration field; the Legendre expansion maps the surface flux onto a sphere without considering the shape of the Janus colloid; while the shape of the Janus colloid can be resolved in the FEM model. Therefore, to study the effect of the shape of the Janus colloid, here, we compare the experimentally measured velocity results with the estimation from the far-field approximation and FEM with and without considering the shape of the Janus colloid.

**FIGURE 2:**
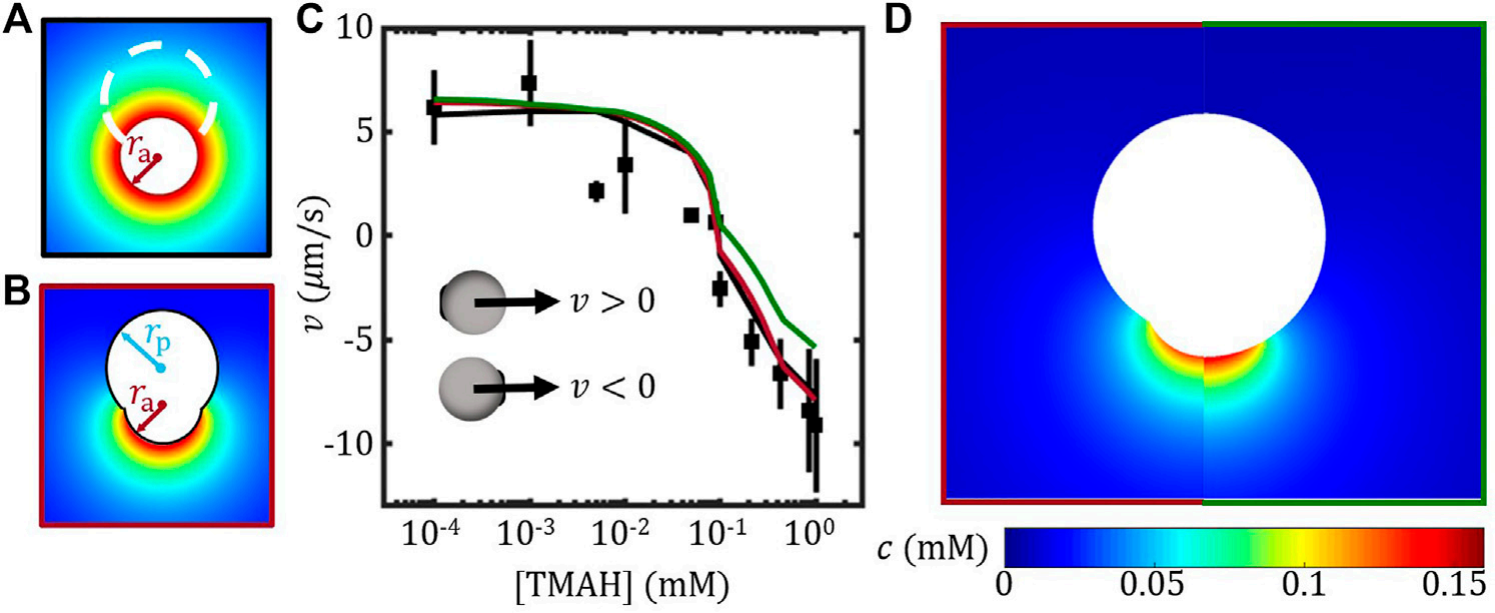
FEM study of the self-propulsion velocity of TPM/haematite Janus colloids. **(A)** Illustration of the far-field approximation **(B)** Construction of the geometric model for the Janus colloids. **(C)** Velocity of Janus colloids *vs.* [TMAH]. FEM results considering the shape effect of the Janus (red) and a spherical Janus (green) are presented together with the experimental results (data points) and far-field approximation (black line) re-plotted from Ref. Zhou et al. (2021). The insets illustrate the definition of velocity direction. **(D)** Comparing the concentration field from FEM between models with (left) and without (right) considering the shape of the Janus colloid.

Figure 2A illustrates the essence of the far-field approximation. The Janus colloid was reduced to a spherical active colloid of a radius set by the active part of the Janus *r*_a_ combined with an imaginary sphere of radius *a* representing the Janus colloid. In this case, the active sphere releases ions isotropically while the imaginary sphere does not affect the ionic concentration field. Thus, the ionic concentration field can be determined using the far-field expression $c_{\text{g}}=\frac{Ja_{\text{s}}^{2}}{D}\frac{1}{r}+c_{\infty }$ with the experimentally-determined surface flux *J*_0_ = 2.2 × 10^–3^ mol/(m^2^ ⋅s). The surface mobility was determined using the experimentally-measured *ζ* potential of the Janus colloids at the centre of the imaginary sphere, i.e. at distance *a* away from the centre of the active sphere. The resulting velocity as a function of the TMAH concentration is plotted as a black line in Figure 2C together with the experimental results from Ref. Zhou et al. (2021). Such an over-simplified model is therefore proved to be sufficient for estimating the self-propulsion velocity of Janus colloids which is not even spherical.

By taking into account the shape of the non-spherical Janus colloid, the FEM model was constructed as illustrated in Figure 2B, so that *r*_a_ and *r*_p_ set the size of the active and inert part of the model while the distance between two centres was determined by ensuring the joining angle *θ*_j_ = 150° for simplicity. In this FEM model, the surface flux of *J*_0_ was applied on the active face, while the surface mobility of the active and the inert face were assigned separately based on the measured *ζ* potential as shown in Figure 1E. The FEM model constructed here is therefore different from that in the Ref. Zhou et al. (2021), which considered a uniformed surface mobility. The resulting velocity by using the current FEM model agrees very well with both the far-field approximation and the experimental results, especially at high [TMAH], see Figure 2C. It suggests that the velocity of this TPM/haematite Janus colloids is mainly determined by the TPM part, presumably due to the joint effect of the high coverage and the high (absolute) surface mobility of the TPM, see Figure 1F. This also explains why far-field approximation is applicable for TPM/haematite Janus colloids.

To study the shape effect using FEM method, the Janus colloids was approximated by a sphere of the same size *a* with the same joining angle of *θ*_j_ = 150°. By applying the surface flux *J*_0_ and the surface mobility of active and inert part separately, the resulting velocity was plotted as green line in Figure 2C. The spherical approximation agreed with the non-spherical model at low TMAH concentration; however, underestimated the velocity as [TMAH] was above 0.1 mM. Given that across the whole range of [TMAH] and in each case (with and without detail shape), the mobility *μ*_h_ and *μ*_TPM_ were the only variables, our result suggests that the spherical approximation is reasonable when the surface mobility contrast between the active and the inert face is small. Presumably, for Janus colloids that far from spherical, the spherical approximation will break down.

Moreover, the concentration field estimated using FEM with and without considering the shape of the Janus colloid are presented in Figure 2D. The high concentration regime of the spherical model extended further out compared to the non-spherical one. According to a recent theoretical study, the pair-wise interaction between two Janus colloids is strongly related to the anisotropic concentration of each colloid (Saha et al. (2019)). The change in concentration field caused by approximating the Janus colloid as a sphere may modify the prediction of the pair-wise interaction. On the other hand, while the far-field approximation works well for calculating velocity, it should fail to explain anisotropic exclusion of passive colloids around a stationary Janus colloid since it wipes out all angular differences in concentration field (Huang et al. (2020)).

### Artificial anisotropy due to Legendre expansion

**FIGURE 3:**
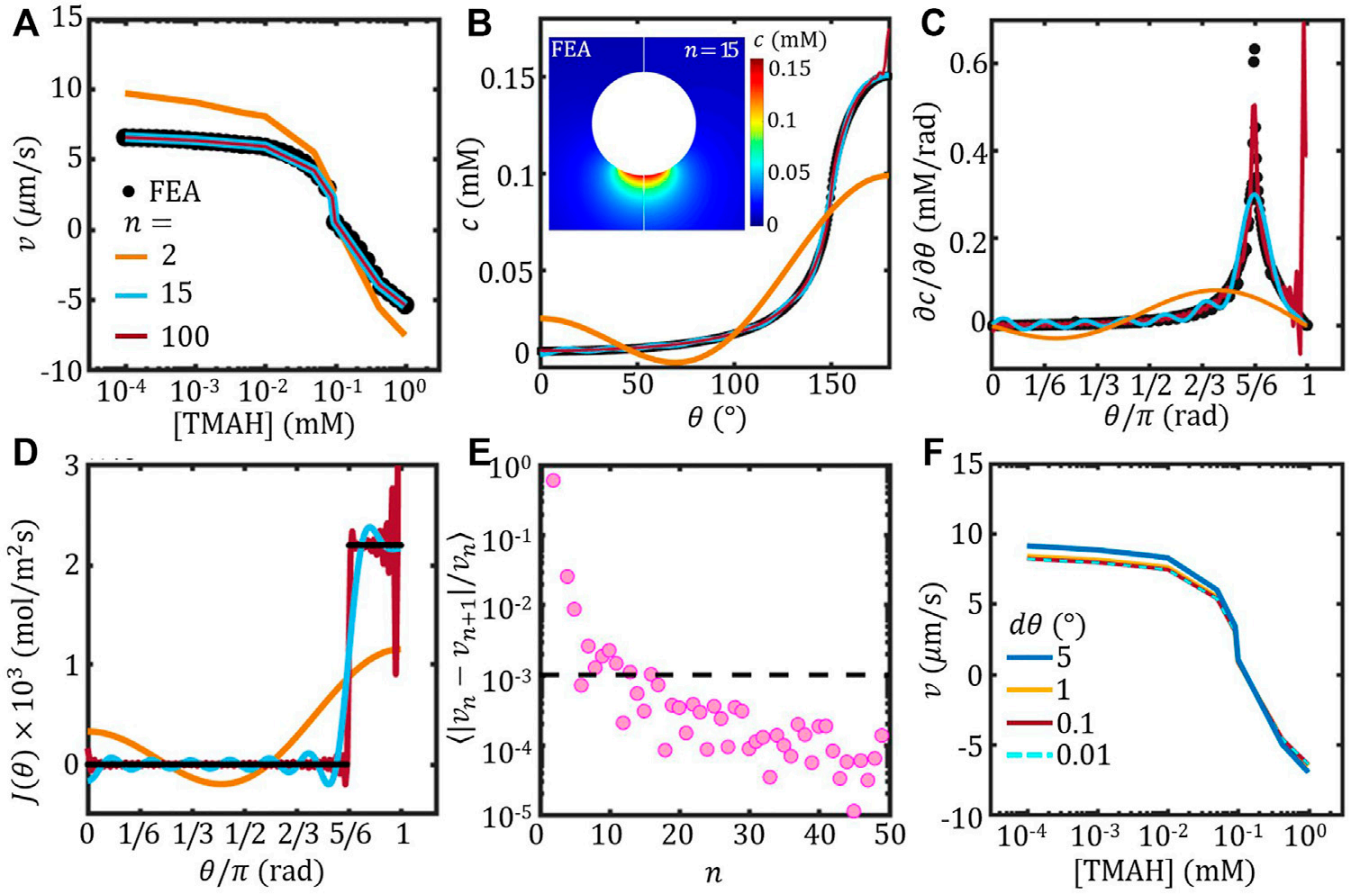
The self-propulsion velocity of a spherical Janus colloid estimated using Legendre expansion. **(A)** Velocity as a function of [TMAH] with different order *n* (lines). The FEM result of a spherical Janus is re-plotted from Figure 2C (circle). **(B**–**D)** Concentration *c* at the surface **(B)**, the corresponding tangential concentration gradient *∂c*/*∂θ* **(C)** and surface flux *J*(*θ*) **(D)** as a function of *θ* evaluated using FEM and Legendre expansion of varying order *n*. Inset of **(B)** shows the comparison of the concentration field of a spherical Janus colloid evaluated using FEM (left) and Legendre expansion to the order of *n* = 15. **(A)**–**(D)** share the same legend. **(E)** The variance of velocity between successive order *n*, ⟨|*v*_*n*_ − *v*_*n*+1_|/*v*_*n*_⟩, as a function of *n* **(F)** Velocity estimated with varying *θ* resolution *dθ*.

The velocity estimated based on the concentration field obtained by Legendre expansion (Eq. 6 of different order *n* was plotted in Figure 3A together with the FEM result re-plotted from Figure 2C. With low order *n*, the Legendre-velocity deviates from the FEM result significantly; however, with increasing *n*, the Legendre-velocity converges to the FEM result. To investigate the anisotropy of the concentration field estimated using Legendre expansion, the angular distribution of the surface concentration, i.e. *c* (*r* = *a*, *θ*) from Eq. 5, was plotted as a function of *θ* in Figure 3B. It shows clearly that with *n* = 2, the concentration distribution was significantly different from the FEM result. The corresponding tangential gradient of the surface concentration *∂c*/*∂θ* further confirms that the anisotropy of the concentration field was distorted at low *n*. With *n* = 15, the wavy angular distribution of the surface concentration and the corresponding tangential gradient recreated the FEM result reasonably well. The inset of Figure 3B further compares the concentration field estimated by FEM and Legendre expansion method with *n* = 15, which shows good agreement.

The artificial anisotropy of the concentration field can be further suppressed by increasing *n*. *c* and *∂c*/*∂θ* estimated with *n* = 100 was plotted in Figures 3B,C, respectively. The wavy feature due to the Legendre expansion was mostly suppressed, except at the very end of the active face (*θ* ≈ *π*). The anomaly around *θ* ≈ *π* was resulted from the imposed surface flux $J\left(\theta \right)=\sum_{0}^{\infty }J_{n}P_{n}\left(\cos\theta \right)$ which fluctuated strongly with high *n* due to the Legendre expansion, as shown in Figure 3D. The angular distribution of the surface flux *J*(*θ*) further shows that the artificial anisotropy of the concentration field was resulted from the Legendre expansion of the surface flux. The fluctuation of *J*(*θ*) was of long wave length with *n* = 2 and deviated from the ideal distribution significantly; and of very short wave length *n* = 100 but broke up at the very end. Therefore, the order of Legendre expansion needs to be chosen carefully depends on the purpose of each study, so that the resulting concentration field can be used to estimate the self-propulsion velocity or to investigate the pair-wise interaction.

For estimating of the self-propulsion velocity, we calculated the variance of velocity between successive order *n*, ⟨|*v*_*n*_ − *v*_*n*+1_|/*v*_*n*_⟩, and plotted the results as a function of *n* in Figure 3E. It shows that the variance is stably lower than 0.1% when *n* > 15. Therefore, the self-propulsion velocity can be considered converged at *n* > 15.

On the other hand, the angular resolution *dθ* can also affect the velocity estimation using Legendre expansion method. The velocity estimated with fixed *n* = 15 and varying the angular resolution is shown in Figure 3F. It shows that with *dθ* < 1° the velocity overlaps onto each other. Note that the computational cost increases with increasing angular resolution, our results suggests that *dθ* = 1° is reasonable.

### Size effect on the self-propulsion velocity

Upon verifying the Legendre expansion and FEM method, we now extend the ionic-diffusiophoresis model to a widely studied model system of Pt/PS Janus colloids. Previous study found that the velocity is proportional to 1/*a*, where *a* is the radius of the Janus colloids (Ebbens et al. (2012)). The authors rationalised this behaviour by considering the neutral diffusiophoresis resulted from the decomposition of H_2_O_2_ into O_2_. It was suggested that the size controlled the concentration field by scaling with the kinetics of the chemical reaction and the diffusivity of the H_2_O_2_, and thus the self-propulsion velocity (Ebbens et al. (2012)). However, Brown et al. has shown that ions should play an important role in the self-propulsion of Pt/PS Janus colloids (Brown and Poon (2014)), yet the alternative mechanisms proposed by Brown et al. did not agree fully with the experimental results (Brown et al. (2017)).

**FIGURE 4:**
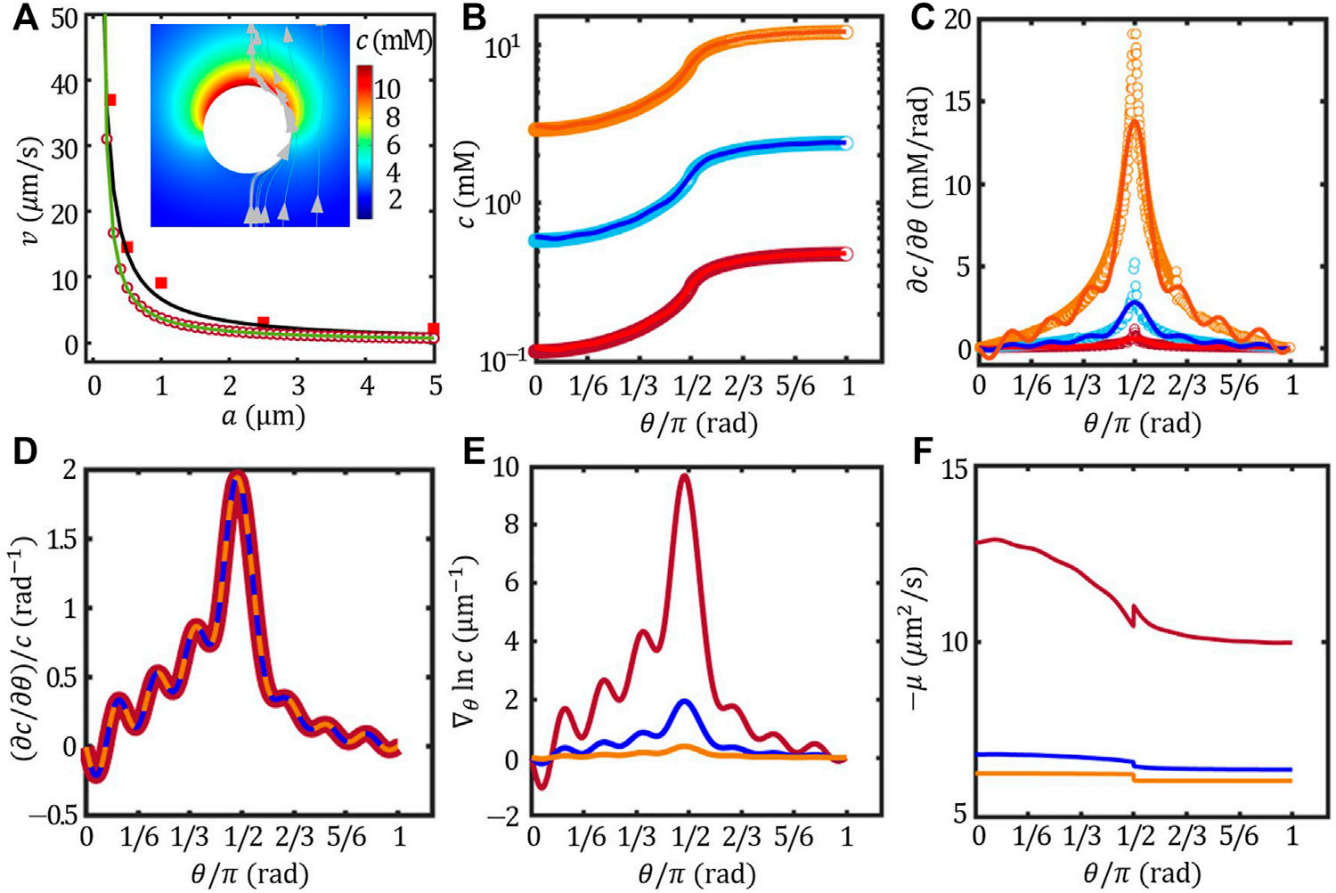
Effect of the colloidal size **(A)** **(A)** The velocity of the Pt/PS Janus colloids as a function of size **(A)** experimental results (red square, re-plotted from Ref. (Ebbens et al. (2012)), far-field approximation (black line), FEM (red circle) and Legendre expansion method of *n* = 15 (green line). The inset presents the concentration field and flow field of the Janus of *a* = 5 μm. The angular distribution of surface concentration *c* **(B)**, the corresponding tangential gradient *∂c*/*∂θ* **(C)** (*∂c*/*∂θ*)/*c* **(D)**, $\nabla_{\theta }\ln c=\left(\frac{1}{a}\frac{\partial c}{\partial \theta }\right)/c$ **(E)** and the opposite of surface mobility − *μ* **(F)**: *a* = 0.2 μm (red), 1 μm (blue) and 5 μm (brown). Circles are obtained from FEM and lines are from Legendre expansion method of *n* = 15.

Here, we hypothesize that Pt catalyzes H_2_O_2_ into H^+^ and OH^−^, and thus the self-propulsion mechanism can be described by the ionic-diffusiophoresis (Eqs 1, 2). Because the *ζ* potential of Pt, PS and the surface activity of Pt in DI water were unknown in Ref. Ebbens et al. (2012), we obtained *ζ*_Pt_ ≈ − 35 mV from Ref (Farniya et al. (2013); Zhang et al. (2017)).; *ζ*_PS_ ≈ − 33 mV from Ref. Zhou et al. (2021); and *J* = 2 × 10^–2^ mol/(m^2^s) from Ref. Brown and Poon (2014). The velocity of Pt/PS Janus colloids estimated using far-field approximation, FEM and Legendre expansion method (*n* = 15) were presented in Figure 4A together with the experimental data re-plotted from Ref. Ebbens et al. (2012). The inset of Figure 4A shows the concentration field of the Pt/PS overlaid with the solvent flow field evaluated using FEM. The flow field shows that the Janus colloid moves towards its inert face (*v* > 0), agreeing with the experimental observation (Ebbens et al. (2012)). Even thought the variation of *ζ* potential with particle size was neglected, our model agreed very well with the experimental results with no fitting parameters.

To understand the good agreement between the ionic-diffusiophoresis model and the experimental results as well as the 1/*a* scaling behaviour of the velocity, we decompose the size effect into two parts:

1) The size effect on the concentrtation field: In the ionic-diffusiophoresis model, the velocity is scaled by the normalised concentration gradient at the surface, i.e., $\nabla_{\theta }\ln c=\left(\frac{1}{a}\frac{\partial c}{\partial \theta }\right)/c$ part in Eq. 6. To investigate the origin of the difference in ∇_*θ*_ ln *c*, the surface concentration *c* and the corresponding gradient *∂c*/*∂θ* of three different size evaluated using FEM and Legendre expansion were presented in Figures 4B,C. Even though the difference in *c* and *∂c*/*∂θ* between sizes was drastic, the ratio $\left(\frac{\partial c}{\partial \theta }\right)/c$ of different sizes collapsed onto each other as shown in Figure 4D. Therefore, the difference in ∇_*θ*_ ln *c* between sizes shown in Figure 4E was originated from the scaling factor $\frac{1}{a}$ . The results shown in Figures 4B–E also demonstrate that the size effect on the concentration field exists even without concerning the kinetics of chemical reaction in detail.

To futher demonstrate the essence of this scaling behaviour, we note that, in the far-field approximation, the concentration field is given by $c_{\text{g}}=\frac{Ja_{\text{s}}^{2}}{D}\frac{1}{r}+c_{\infty }$ . When *c*_*∞*_ is small, ∇ ln *c* is readily given by 1/*a* at the surface of the colloids. When combined with the fact that *ζ*_Pt_ ≈ *ζ*_PS_, it may explain why, as oversimplified as it is, the far-field approximation can match the experimental result nicely.

2) The size effect on the surface mobility: The velocity is scaled by the surface mobility *μ* which is size-dependent, see Eq. 2. The distribution of the opposite of *μ* as a function of *θ* is shown in Figure 4F. Generally, − *μ* of the inert face (*θ* < *π*/2) was larger than that of the active face (*θ* > *π*/2), even though *ζ*_Pt_ ≈ *ζ*_PS_ in this case. This is because the mobility also depends on the local Debye length, and thus the concentration field at the colloidal surface *c*(*θ*). Figure 4F demonstrates that the size-dependent surface mobility also contributes to the size effect on the self-propulsion velocity.

**FIGURE 5:**
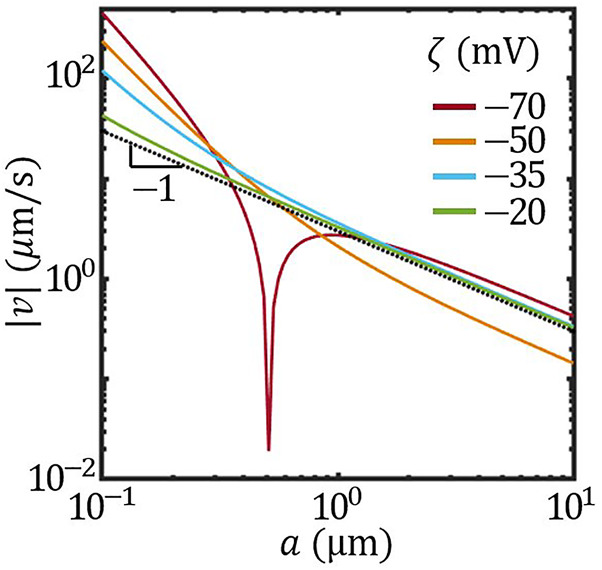
The size-dependent speed of Janus colloids with different overall *ζ* potential (solid lines). Dotted black line plots |*v*|∝ 1/*a*.

Furthermore, when the size effect on the surface mobility overwhelms that on the concentration field, the scaling behaviour of the velocity can be altered beyond 1/*a*. According to Eq. 2, this can be realized by varying the overall *ζ* potential of the colloid. Figure 5 presents the scaling behaviour of the speed |*v*| *vs*. *a* with different overall *ζ* potential. With *ζ* = −20 mV, the scaling behaviour of |*v*| was very close to |*v*|∝ 1/*a*. As *ζ* potential decreased, taking −25 mV and −50 mV as examples, the scaling behaviour gradually deviated from 1/*a*. Further lowering the overall *ζ* to −70 mV led to the reversal of the velocity at *a* ∼ 0.5 μm. This size-induced velocity reversal is reminiscent of the transition between active-passive attraction and repulsion reported previously (Zhou et al. (2021)).

Figures 4, 5 indicate that, in the ionic-diffusiophoresis model (Eqs 1, 2), the size of Janus colloid affects the self-propulsion velocity by influencing not only the distribution of the concentration field but also the magnitude of the surface mobility. This is because the mobility determined by Eq. 2 considers a finite Debye length comparable to the size *a*, which is true in experiments especially in solutions of low ionic strength. Thus, our study provides a possible explanation for the observed size-dependent velocity.

## Conclusion

We studied the velocity of Janus colloids depending on the anisotropic concentration field, considering the effect of the shape, theoretical treatment using Legendre expansion and eventually the colloidal size, within the framework of ionic-diffusiophoresis. Compared with the experimentally measured velocity of TPM/haematite Janus colloids, the spherical approximation is reasonable if the surface mobility contrast between the active and the inert faces is modest. Raising the order of the Legendre expansion can minimise the artificial anisotropy it generates; however, greater order may cause distortion of the concentration field at the zenith of the active face. Therefore, the order of the expansion needs to be chosen carefully depending on the purpose of the study. Our work rationalises the Legendre expansion method and provide guidelines for its further application. The ionic-diffusiophoresis model was futher applied to investigate the size-dependent velocity of Pt/PS model system. Our model agrees very well with the 1/*a* scaling behaviour observed previously, with no fitting parameters. We also showed that the colloidal size can reverse the self-propulsion velocity depending on the overall *ζ* potential of the colloids. By analysing the angular distribution of the concentration field and the surface mobility of Janus colloids with different sizes, we attribute the size effect on the velocity to the size effects on both th concentration field anisotropy and the surface mobility. Our results provide not only new insights into the self-propulsion mechanism of chemically-driven active colloids but also a new way to control their dynamics.

## Data Availability

The original contributions presented in the study are included in the article/Supplementary Material further inquiries can be directed to the corresponding author.
