# Supplementary material for: Estimating the velocity of chemically-driven Janus colloids considering the anisotropic concentration field
Source: Front Chem. 2022 Aug 12;10:973961. doi: 10.3389/fchem.2022.973961 (PMC9411653; doi:10.3389/fchem.2022.973961)
Supplement: Supplementary file 1 [file DataSheet1.PDF]

Supplementary Material

1 ELEMENTAL MAPS OF THE JANUS COLLOID

The scanning electron microscopy (SEM) and the energy dispersive X-ray Analysis (EDX) were employed to indentify the active (catalytic) face of the TPM/haematite Janus colloids. The EDX spectrum imaging was conducted with a Zeiss Gemini SEM 300 system. The resulting elemental maps are shown in Figure S1. The results confirm that, in the SEM images, the white cube-shaped part of the Janus colloid is the haematite colloid, i.e. the active part.

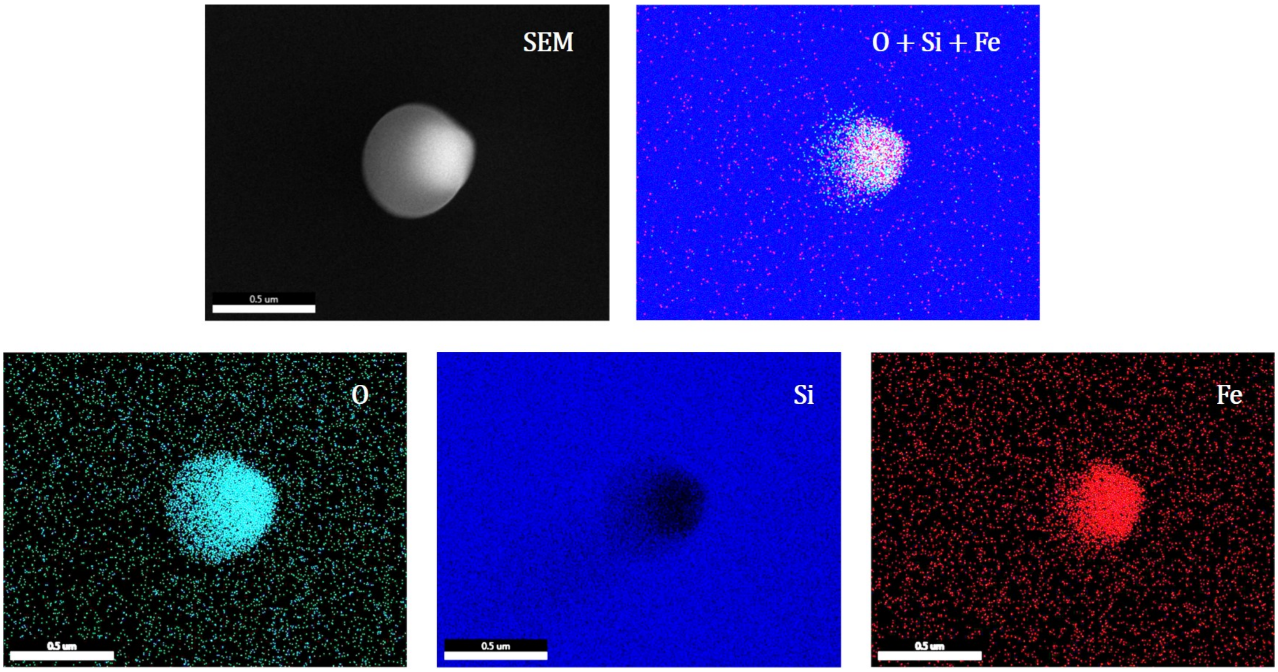

Figure S1. The SEM image and EDX elemental maps of TPM/haematite Janus colloids.
